# Kinetics of the Release of Nicotinamide Absorbed on Partially Neutralized Poly(acrylic-co-methacrylic acid) Xerogel under the Conditions of Simultaneous Microwave Heating and Cooling

**DOI:** 10.3390/gels7040193

**Published:** 2021-10-31

**Authors:** Jelena D. Jovanovic, Borivoj K. Adnadjevic

**Affiliations:** 1 Institute of General and Physical Chemistry, University of Belgrade, Studentski trg 12–16, 11001 Belgrade, Serbia; 2 Faculty of Physical Chemistry, University of Belgrade, Studentski trg 12–16, 11001 Belgrade, Serbia; borivoj@ffh.bg.ac.rs

**Keywords:** xerogel/hydrogel, kinetics, microwave heating, nicotinamide, release

## Abstract

The kinetics of release of nicotinamide (NIAM) that was absorbed on partially neutralized poly(acrylic-co-methacrylic) (PAM) xerogel/hydrogel, under the conditions of simultaneous microwave heating and cooling (SMHC) were examined. The kinetics curves of NIAM release into an aqueous solution at temperatures of 308–323 K were recorded. By applying the model-fitting method (MFM), it was found that the kinetics of NIAM release can be modeled by a kinetic model of a first-order chemical reaction. The values of the release rate constants (*k_M_*) at different temperatures were calculated, and their values were found to be within the range 8.4 10^−3^ s ^−1^−15.7 10^−3^ s^−1^. It has been established that the Arrhenius equation was valid even in the conditions of SMHC. The values of the kinetic parameters (activation energy (Ea) and pre-exponential factor (A) of the NIAM release process adsorbed on PAM xerogel/hydrogel were calculated as follows: Ea = 25.6 kJ/mol and ln (A/s^−1^) = 5.21. It has been proven that the higher value of the rate constant at SMHC in relation to CH is not a consequence of the overheating of the reaction system or the appearance of “hot-points”. The values of change of the enthalpy of activation (ΔH*) and the change of entropy of activation (ΔS*) were calculated as follows: ΔH* = +23.82 kJ/mol and ΔS* = −201.4 J/mol K. The calculated higher values of the kinetic parameters and thermodynamic parameters of activation are explained by the formation of a specific activated complex under SMHC, whose structure and degree of order are different than in the one formed under CH.

## 1. Introduction

Nicotinamide (pyridine-3-carboxamide) is an amide form of niacin and belongs to the B-vitamins group. Nicotinamide is water-soluble vitamin which is not deposited in the body. In spite of that, both niacin and nicotinamide are similar in their roles as vitamins, but they differ in their pharmacologic indications and effects. The most common synonyms for nicotinamide are vitamin B3, niacinamide, nicotinic acid amide, nicotinic amide and vitamin PP. Nicotinamide is chemically part of two nucleotide coenzymes: nicotinamide adenine dinucleotide (NAD+) and nicotinamide adenine dinucleotide phosphate (NADH) [1]. They are involved in many important biological oxidation-reduction reactions, among which is the production of adenosine triphosphate (ATP) [2].

The first clinical use of nicotinamide was for the treatment of the disease pellagra [3]. There are numerous studies that have shown the beneficial effect of nicotinamide when included in therapy for many diseases: apoptosis [4], tuberculosis, human immunodeficiency virus (HIV) [5], and diabetes [6]. A large study found that taking nicotinamide can reduce the risk of some types of cancer, namely skin cancers and nonmelanoma skin cancers [7]. Nicotinamide may protect high-risk individuals against some forms of skin lesions, particularly in patients with sun-damaged skin. Nicotinamide is used for treating skin conditions such as acne, rosacea and rough, scaly patches [8]. The effects of oral or topical nicotinamide are associated with its photo-immune-protectiveness, which is linked to its support for DNA repair by preventing post-UV exposure declines in cellular energy or the repletion of energy to irradiated cells [9]. It contributes, on several pathways, to this enhanced repair of UV-induced DNA damage [10]. Skin cancer chemoprevention is attributed in part to reductions in inflammatory macrophages [11]. The effects of topical nicotinamide on inflammatory skin conditions are attributed to its sebo-suppressive and anti-inflammatory properties [1]. Nicotinamide demonstrates antiaging effects, since increases collagen production and the epidermal proteins, which has been proven to result in significant decreases in wrinkles and skin roughness [12]. The existing literature and clinical data specify that nicotinamide is an inexpensive and safe as drug with numerous beneficial effects, which make it appropriate to be used both as a topical and oral drug without major adverse effects [13].

It has been known for a long time that products may be applied to the skin for either local or systemic effects [14]. Hence, it can be expected that NIAM would be an excellent candidate for transdermal drug delivery (TDD). When compared to other traditional (oral and parenteral) approaches to drug delivery, TDD may offer considerable advantages, such as long-term releasing, lower toxicity, avoidance of passing the liver, etc. [15]. The development of new transdermal devices presents an integral part of the current general and sustaining delivery of drugs. Therefore, the predictability and reproducibility of their release kinetics and their bioavailability should be objects of permanent improvement. There are a number of successful commercially available TDD systems at present, but the need to extend their operating parameters so that more sustainable and variable delivery regimes can be achieved still exists. In regard to the potential applicability of NIAM, the development of transdermal matrix capable of successfully delivering it is of paramount importance.

Recently, M. Kitaoka developed oil-based microemulsion formulations with an aim to enhance the transdermal delivery efficiency of nicotinamide. The developed monoolein-based microemulsion was transparent and stable, suggesting that it is a promising formulation for a transdermal nicotinamide delivery [16]. A hybrid system based on nicotinamide and nanoparticles (NPs) encapsulating the immunosuppressant tacrolimus (FK506), which hardly penetrates into and through the skin, for facilitating percutaneous delivery, was developed [17]. Drug penetration through to the relevant skin layers is one of the major scientific concerns for the treatment of atopic eczema. El-Menshawea et al. prepared a new formulation based on positively charged ethosomes as drug carriers. In vivo testing of the thermosensitive positively charged ethosomal gel of nicotinamide on induced eczema in animal models revealed an enormous recession of inflammatory reaction associated with eczema, in addition to a significant reduction of corneocytes maturation [18].

Hydrogels have been widely used for TDD due to their ability to adhere to human skin and to provide a water environment so that drug release may occur once attached to the skin [15]. Hydrogels are three-dimensional (3-D) network polymeric structures based on crosslinked hydrophilic polymers that are able to absorb and retain in their internal structure a high amount of water or aqueous solutions, without dissolving or losing their structural integrity. This phenomenon of absorbing a high amount of water and other fluids (>100% their weight) in a short time is well known, as swelling and is one of the most striking features of hydrogels [19]. The swelling potential of hydrogels and their swelling kinetics depends on their structural properties as well as on the external medium [20]. Due to their outstanding properties, hydrogels have been used in a wide range of biological, medical and pharmaceutical applications, as well as in other fields, such as in personal care products, agriculture, environmental protection, waste water treatment, water purification, etc. [21]. Nevertheless, the most attractive and powerful applications for hydrogels are tissue engineering [22] and controlled-release systems for targeted delivery of drugs to specific areas of the body, which can be achieved in few ways, among which is transdermal delivery [15].

Hydrogels based on poly(acrylic acid) and their copolymers, complexes, interpenetrating networks or grafted networks have been often used as carriers in drug release systems in recent years. Despite the great applicative potential for poly(acrylic acid) hydrogels and opportunities for their modification with different types of co-monomer units, some of the least-used hydrogels are based on copolymers of poly(acrylic-co-methacrylic) acid [23].

Regardless its remarkable scientific and applicative significance, the kinetics of release of NIAM absorbed on the xerogel/hydrogel has been, to our best knowledge, investigated in very few papers. Karadag et al. used hydrogels based on acrylamide and itaconic acid, loaded with nicotine, nicotinic acid, nicotinamide and nikethamide to investigate their swelling kinetics in vitro. Swelling kinetics have been described for all of the investigated drugs as a non-Fickian type of diffusion and it was determined that the swelling rate increased in accordance with the following order: nicotine > nikethamide > nicotinamide > nicotinic acid [24,25]. Songkro et al. prepared hydrogels based on different structural unities using poly(acrylic acid), (commercial “Carbopols”), hydroxypropylmethyl cellulose (HPMC), sodium carboxymethyl cellulose (SCMC) and methylcellulose (MC) loaded with nicotinamide. Their mucoadhesive properties and in vitro NIAM release from all the hydrogel formulations have been investigated. The anionic polymers, Carbopol, and SCMC have been found to be more appropriate by physical properties (appearance, pH, viscosity and adhesiveness), mucoadhesive properties and release rates of NIAM than the neutral polymers HPMC and MC [26]. Dobić et al. prepared hydrogels based on 2-hydroxyethyl methacrylate, poly(ethylene glycol), dimethacrylates and itaconic acid by free radical crosslinking copolymerization. The synthesized hydrogels indicate acceptable cytocompatibility. The authors investigated in vitro release of nicotinamide and nicotinic acid by fitting the dissolution profiles to various mathematical models. The best fit was obtained for first-order kinetics [27]. The hybrid system (FK506 NPs-NIC) consisting of nanoparticles (NPs) based on the self-assembling of hyaluronic acid and cholesterol (HA-Chol NPs) containing nicotinamide (NIAM) for tacrolimus (FK506) release were prepared with the aim of evaluating the potential of tacrolimus for the treatment of psoriasis. In vitro permeation through the psoriatic skin was carried out, and the results revealed that the combination of NPs with NIAM exhibited a significant synergistic effect on FK506 deposition within the psoriatic skin (3.40 ± 0.67 μg/cm^2^) and penetration through the psoriatic skin (30.86 ± 9.66 μg/cm^2^). The results support that the investigated hybrid system in combination with NIAM is promising for the treatment of psoriasis [28]. In the paper of Djajic et al., the isothermal kinetics the release of NIAM that has been absorbed on PAM xerogel/hydrogel under the conditions of conventional heating (CH) was investigated. Based on the obtained results in that paper, it was concluded that (a) NIAM absorbed on PAM under the conditions of CH was not released entirely (about 60%), (b) the kinetics of NIAM release can be mathematically modeled by the reversible first order chemical reaction model and (c) the activation energy (Ea) and pre-exponential factor (A) of the release are higher than the Ea of the absorption. The kinetics of NIAM release was controlled with the rate of distribution of NIAM between the hydrogel and the surrounding solution [29].

Nowadays, microwave heating (MWH) is acknowledged as one of the non-conventional, energy-efficient sources of energy. Microwave heating considerably accelerates the rate of chemical reactions and physico-chemical processes and leads to higher yields of products, frequently with improved properties and better selectivity. For all these reasons, the application of microwaves merits more attention and has become an area of increasing fundamental and practical interest [30].

Bearing in mind that MWH significantly accelerated chemical reactions and physico-chemical processes, in this paper, to the best of our knowledge, for first time, the kinetics of NIAM release under the conditions of simultaneous microwave heating and cooling (SMCH) are investigated, with the aim of finding and explaining the effects of MWH on the kinetics of release of drugs absorbed on hydrogels.

## 2. Results and Discussion

**Figure 1:**
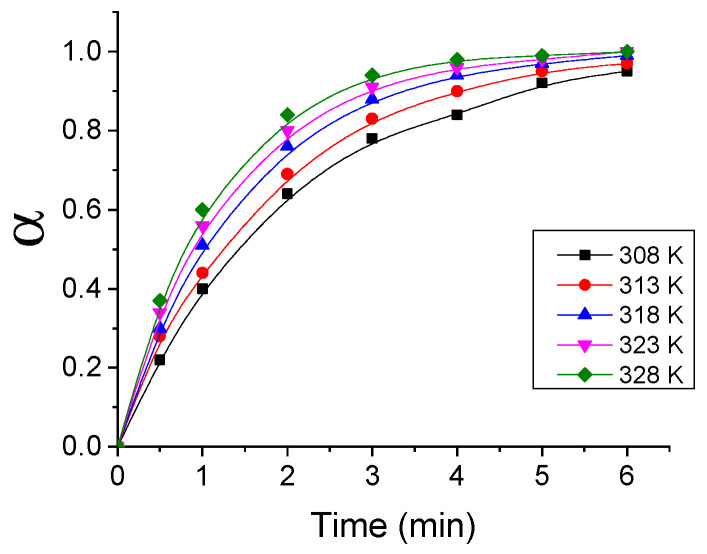
Isothermal kinetic curves for NIAM release under SMHC.

The isothermal kinetic release curves of NIAM absorbed on PAM xerogel/hydrogel under the conditions of microwave heating and simultaneous cooling are shown in Figure 1.

The isothermal kinetic curves of NIAM release show three characteristic shapes of the increase of NIAM concentrations in solutions, with release durations. Within short release times (t ≤ 1 min), NIAM concentration increases almost linearly with the increase in time. Further prolongation of the release time leads to a slowdown of the increase of concentration with time (convex increase). At the end of the release process, the concentration of NIAM achieves its maximum value (Cmax = 1.2 g/L) and does not change with further increase in time (plateau).

The increase in the temperature of the release leads to the increase in the slope of the linear change in concentration with time, and to a decrease in the time necessary to achieve maximal concentration of released NIAM, which indicates that the rate of NIAM release is accelerated with the increase in temperature, and that it is a thermally activated process. If we compare kinetic release curves of NIAM under the conditions of isothermal CH with the kinetic release curves obtained under SMHC, it can be concluded that SMHC (a) leads to complete release of NIAM (b) does not change the shape of the kinetic and (c) considerably reduces the time required for NIAM release (from 120 min to 6 min).

**Figure 2:**
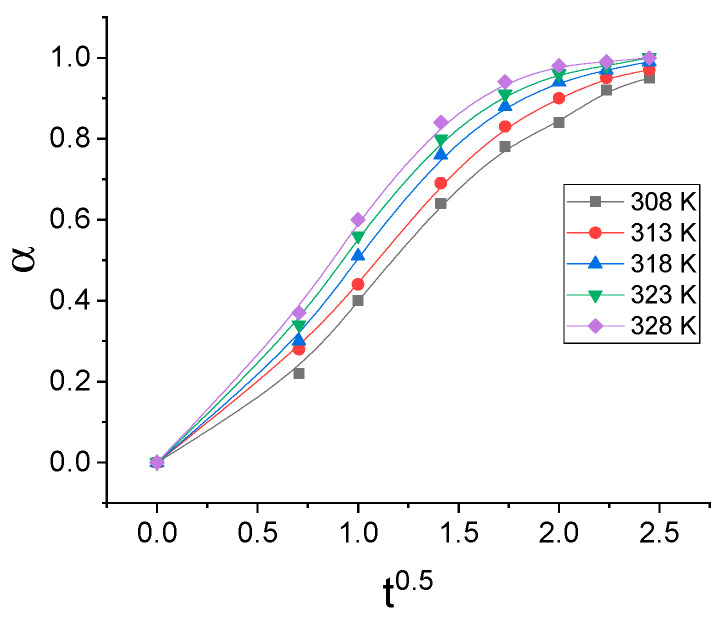
Isothermal dependence of α on t^0.5^.

The release kinetics of drugs from the polymer matrix can be kinetically modeled by a series of equations such as Higuchi, Peppas, Hixon–Crowell, Hopfenberg, Weibill, etc. [31]. Having in mind the assumptions of different mathematical models of drug release kinetics, the possibility of describing the release kinetics of NIAM by applying Higuchi’s and Peppas’s models was investigated. In case the kinetics of NIAM release can be described by Higuchi’s model, the dependence of α on t^0.5^ should be a straight line. Figure 2 shows the dependence α on t^0.5^.

**Figure 3:**
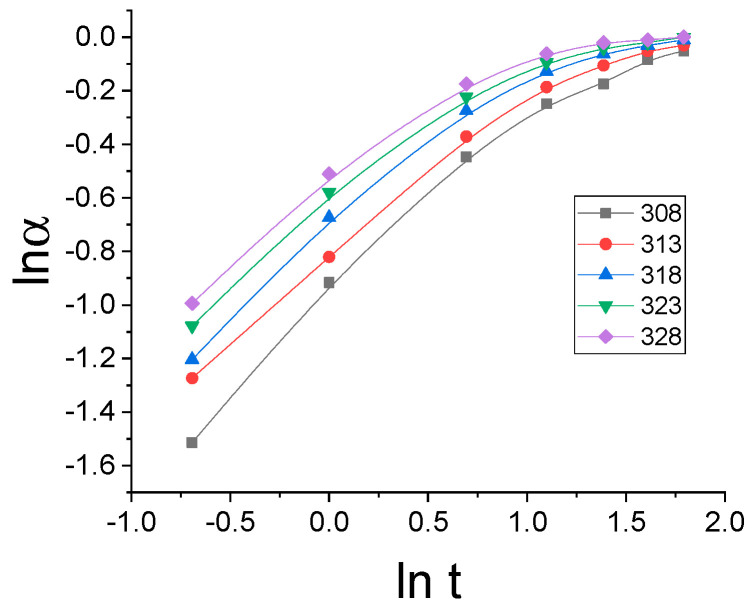
Isothermal dependence of *lnα* on *lnt*.

It can be seen from the Figure 2 that the dependence α on t^0.5^ deviates significantly from the straight line, which indicates that the release kinetics of NIAM cannot be modeled by Higuchi’s model. In case the kinetics of NIAM release can be described by Peppas’s model, the dependence of lnα on lnt should be a straight line. Figure 3 shows the dependence lα on lnt.

It can be seen from the Figure 3 that the dependence of lnα on lnt deviates significantly from the straight line, which implies that the release kinetics of NIAM cannot be modeled by Peppas’s model either.

**Figure 4:**
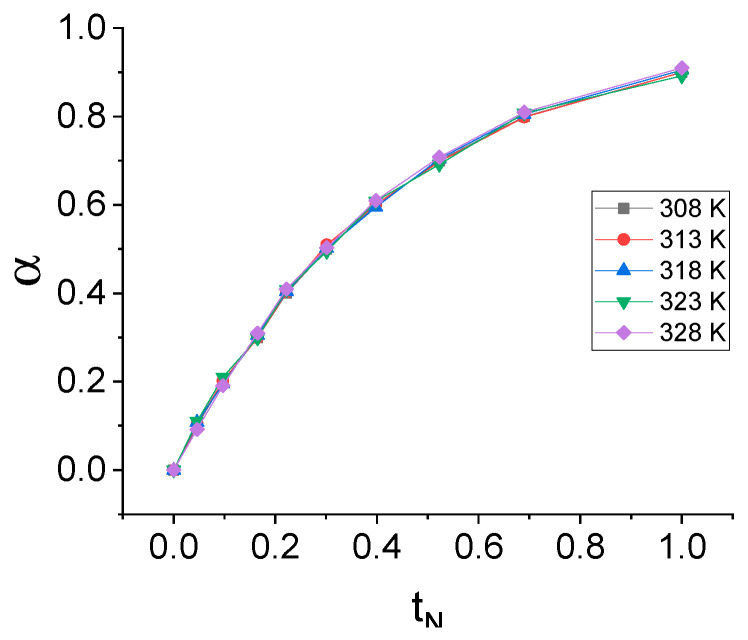
Isothermal normalized conversion curves of NIAM release.

Due to the impossibility of describing the release kinetics of NIAM with the most- commonly used models, the determination of the kinetic model was performed with a model-fitting model [32]. In order to determine the kinetic model of NIAM release under the SMHC, the isothermal kinetic release curves were transformed into normalized conversion curves, ie. α vs. t_N_. The isothermal normalized conversion curves of NIAM release are shown in Figure 4.

The isothermal normalized conversion curves of NIAM release under SMHC have identical shapes at all of the investigated temperatures, which indicates a unique kinetic model of NIAM release that is independent of temperature. By using the model-fitting method [32], it was concluded that the isothermal kinetics of NIAM release under SMHC can be modeled by a kinetic model of a first-order chemical reaction:

(1) $$\alpha =1-exp\left(-k_{M}t\right)$$

where *k_M_* is the model’s rate constant of NIAM release.

The Equation (1) was linearized into the linear form:

(2) −ln(1−α)=t

**Figure 5:**
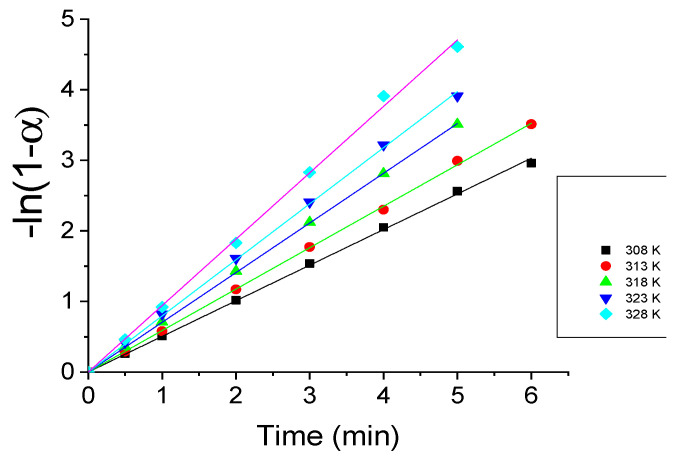
Isothermal dependences of −*ln*(1 − *α*) on time.

The isothermal dependences of −*ln*(1 − *α*) on time are shown in Figure 5.

The isothermal dependences −*ln*(1 − *α*) on time are straight lines at all of the investigated temperatures, which confirms the correctness of the chosen kinetic model of release and allows the calculation of the value of *k_M_*. The isothermal values of *k_M_* are shown in Table 1.

As can be seen from the data shown in Table 1, the *k_M_* values obtained under the SMCH increase with temperature, as in the case for CH. The values of *k_M_* for SMCH conditions are significantly higher than those obtained under CH. By analyzing the increase in the value of *k_M_* with temperature, it can be concluded that once again, in the case of SMCH, the increase in the value of *k_M_* can be described by the Arrhenius equation. Based on this, using the Arrhenius equation, the values of the kinetic parameters of the release (*E_a_* and *lnA*) were calculated (columns 3 and 4 in Table 1) and are as follows: *E_a_* = 25.6 kJ /mol and ln(A/s^−1^) = 5.21. The values of the kinetic release parameters under SMHC conditions are significantly higher than the corresponding ones for isothermal CH.

The acceleration of chemical reactions and physico-chemical processes under the conditions of microwave heating (MWH) is most often explained by the so-called thermal effects (overheating, hot spots, selective heating and non-thermal specific microwave effects [33].

The calculated values of *k_M_* and kinetic parameters (*E_a_*, *lnA*) in CH and SMHC make it possible to determine objectively if there exist the so-called hot-spot points when performing experimental measurements in SMHC conditions. Namely, if it is assumed that the values of kinetic parameters (*E_a_*, *lnA*) in SMHC are identical to the values of kinetic parameters (*E_a_*, *lnA*) in CH, then based on the knowledge of *k_M_* and the established validity of the Arrhenius equation in SMHC conditions, the temperature value can be calculated (T*) in the reaction mixture corresponding to a certain value of *k_M_* (*T*).

(3) $$T^{*}=\frac{E_{a}\cdot T}{E_{a}-R\cdot T\cdot lnZ}$$

where: $Z=\frac{k_{M}^{SMHC}}{k^{CH}}$ and *R* is universal gas constant.

Table 2 shows the calculated values of T* at the investigated temperatures under SMHC conditions.

Since the values of T* are unrealistic and significantly higher than the realistically measured temperatures in the reaction system (261–387 K), it can be claimed, with a high degree of certainty, that the increase in isothermal rate constants obtained under the conditions of SMHC in comparison to their values obtained under CH conditions is not due to the specific (non-thermal) action of the microwave field on the kinetics of the NIAM release reaction or the existence of hot spots.

The increase in the amount of NIAM released in the release rate constant and in the kinetics parameters (Ea and *lnA*) under the SMHC conditions in comparison to isothermal CH conditions is most probably due to the specific effects of the MW field on the release kinetics. Knowing that the release kinetics of NIAM in SMHC can be mathematically described by the kinetic model of the first order chemical reaction, and that it is thermally activated (in other words, the Arrhenius equation is valid), the values of change in enthalpy of activation (ΔH*) and change in entropy of activation (ΔS*) in SMHC and CH were calculated, with the intention to explain the influence of MW fields on kinetics. The calculation of the ΔH* and ΔS* values was performed using the Eyring–Polanyi equation [34]:

(4) $$k_{M}=\frac{k_{b}T}{h}\;exp\left(\frac{\Delta S^{*}}{R}\right)exp\left(-\frac{\Delta H^{*}}{RT}\right)$$

where *k_b_* is Boltzman’s constant, *h* is Planck’s constant, *R* is gas constant,

$\Delta H^{*}=H^{*}-H_{0}$, $\Delta S^{*}=S^{*}-S_{0}$, H* is enthalpy of the activated complex, S* is entropy of activated complex and *H*_o_ and *S*_o_ are enthalpy and entropy of the reactants.

The linearized form of Equation (4) can be written as follows:

(5) $$ln\left(\frac{k_{M}}{T}\right)=ln\left(\frac{k_{B}}{h}\right)+\left(\frac{\Delta S^{*}}{R}\right)-\left(\frac{\Delta H^{*}}{R\;T}\right)$$

The plot of ln(*k_M_*/*T*) vs. reciprocal temperature (1/*T*) provides a straight line with the slope: ΔH*/*R* and the intercept is: *ln*(*k_B_*/*h*) + ((ΔS*)/*R*).

**Figure 6:**
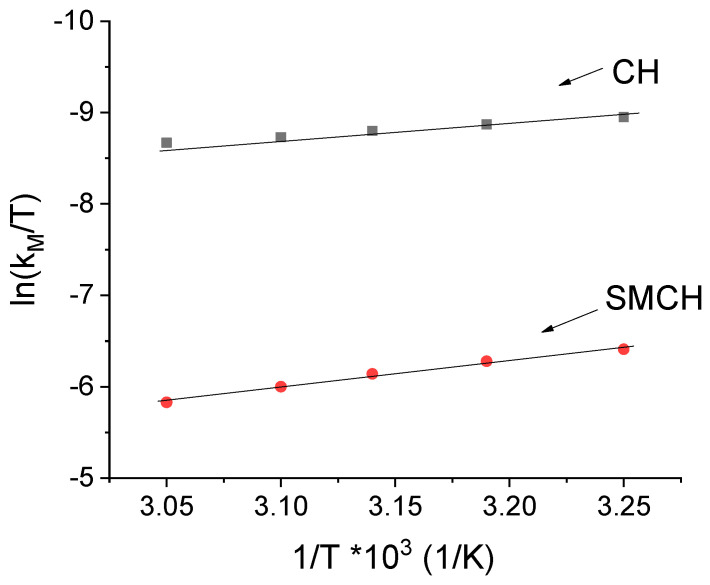
Dependence of ln (*k_M_*/*T*) on (1/*T*) for the case of SMHC and CH.

Figure 6 shows the dependence of ln (*k_M_*/*T*) on (1/*T*) for the case of SMHC and CH.

The dependences of ln (*k_M_ T*) on 1/*T*, for both CH and SMHC, are straight lines, in the entire temperature range. The values ΔH* and ΔS* were calculated from the slope and the intercept of the above linear dependences. The calculated values of ΔH* and ΔS* are shown in Table 3.

As can be seen from the results shown in the Table 3, in the case of SMHC conditions, a significant increase in the values of ΔH* and ΔS* in comparison to their values under CH occurs. It is well-known that the magnitudes of H* and S* are in relationship with the transition-state structure. The values of H* correspond to the energy required for bond reorganization of reacting molecules during active complex formation, whereas S* is a measure of the degree of order of the formed active complex. In cases when translation, rotational and vibrational degree of freedom are lost during the formation of the active complex, there will be a decrease in the value of entropy of activation (S*). Conversely, if the number of the degrees of freedom increase during the formation of the active complex, the value of entropy of activation increases also. Since the values of ΔH* have positive signs, whereas the values of ΔS* have negative signs, based on calculated values of ΔH* and ΔS* under SMHC and CH, it can be concluded that SMHC leads to the formation of an active complex with a structure of completely different chemical bonds then the ones formed under CH conditions. The energy required for bond reorganization in molecules of absorbed NIAM on xerogel/hydrogel to form an active complex is significantly higher in SMHC. That is why the Ea required for NIAM release under SMHC is significantly higher than the one under CH conditions. The value of S* that is obtained for activation when used SMHC is higher than the one obtained by applying CH, which is an indication of the increase in the degree of freedom in an activated complex under SMHC in comparation to CH. The increase in the degree of freedom under SMHC is the fundamental cause of the significant increase in *lnA* under SMHC conditions. Therefore, a significant increase in both release degree and release rate of adsorbed NIAM under SMHC in comparison to CH is due to the formation of an active complex whose structure and mobility of atoms is significantly different from the structure of the active complex formed under CH conditions. Actually, the active complex formed under the SMHC has a different order of positions of atoms and bonds as well as a lower degree of ordering and a higher mobility of atoms in comparison to the active complex that is formed under CH conditions.

Knowledge of the values of NIAM kinetic release parameters under SMCH and CH conditions enables, in accordance with the selective energy transfer (SET) model of activation of reactant molecules for chemical reaction [35,36], a deeper understanding of the mechanism of activation and the structures of the active complex and release reaction. The basic assumption of the SET model is that there is a correlation relationship between the values of kinetic parameters determined at different experimental conditions, the so-called interactive compensation effect, which is given by the expression:

(6) $$lnA_{i}=a+bEa_{i}$$

where *Ea_i_* and *lnA_i_* are the values of *Ea_i_* and *lnA_i_* at different experimental conditions.

If we analyze the relationship between the values of *Ea_i_* and *lnA_i_* obtained under SMCH and CH conditions, which are given in Table 4, the following expression can be obtained:

(7) $$lnA_{i}=-10.36+0.607Ea_{i}$$

Based on the calculated value of the parameter *b* = 0.607 mol/kJ, the values of: wave number of resonant frequency (ʋ), the activation quantum (ε) and the anharmonicity factor (x) are calculated. Table 2 shows the values of ʋ, ε and x

Therefore, the formation of an active complex of the reaction of release of absorbed NIAM from PAM xerogel/hydrogel into water occurs due to the selective (resonant) transfer of the required amount of energy (Ea) from the reaction system to a certain damped oscillator (resonant oscillator) of the molecules of absorbed NIAM with a wave number of ʋ = 274 cm^−1^. The twisting vibration ((NH_2_)τ) of the NH2 group in the NIAM molecule corresponds to that resonant frequency [37]. Resonant energy transfer from the reaction system to the absorbed NIAM molecule leads to changes in the distribution of localized energies by the bonds in the NIAM molecule and the rate of internal energy transfers between them. Therefore, there is a significant increase in the energy localized on the (NH_2_)τ, which creates the conditions for the release of absorbed NIAM molecules. The release of NIAM molecules from the absorbed state leads to a significant increase in the mean rate of internal energy transfers, as a result of which the value of the pre-exponential factor increases.

## 3. Conclusions

Based on the results obtained in this paper, it can be concluded that under the conditions of SMHC, the following happens: (a) complete release of NIAM absorbed on PAM xerogel/hydrogel; (b) the release rate of NAIM is 12–17 fold higher than the release rate from the same hydrogel under the CH conditions; (c) the release kinetics can be described by a first order chemical reaction model; (d) the increase in the rate of NIAM release is not a consequence of overheating in the rection system; (e) the values of kinetic parameters (Ea and *lnA*) are higher than the ones found for the same process occurring under CH conditions; (f) the increase in kinetics parameters is a consequence of the specific effects of the MW field; (g) the active complex is formed, whose structure and mobility of atoms are different from those of a complex formed under CH conditions.

## 4. Materials and Methods

### 4.1. Materials

Monomers, acrylic acid (95%) (AA) and methacrylic acid (MA) were provided by Merck KGaA, Darmstadt, Germany. The cross-linker, N,N′-Methylenebisacrylamide (p.a) (MBA) was obtained from Aldrich Chemical Co., Milwaukee, USA. The initiator, 2,2-azobis-[2-(2-imidazolin-2-il)-propane] dihydrochloride (VA-044) was purchased from Wako Pure Chemical Industries, Osaka, Japan. Sodium carbonate (Na_2_CO_3_) (p.a) was supplied from Merck KGaA, Darmstadt, Germany. Nicotinamide (>98%) was purchased from Fluka AG, Switzerland. All chemicals were used as received. Bidistilled water was used throughout all the experiments.

### 4.2. Synthesis of Poly(acrylic acid-co-methacrylic acid) Xerogel

The synthesis of poly(acrylic acid-co-methacrylic acid) (PAM) xerogel was performed by a previously described process based on crosslinking free-radical copolymerization that was thoroughly described by [29], which, in brief, consists of the following: A 20 wt% aqueous solution AA and MA (3/1 mol/mol) was placed in a thermostatic reactor and stirred well (400 rpm) for 15 min to ensure homogeneity. The MBA solution (1 wt%) was added to the monomers solution in an amount of 0.42 mol% of the monomers, and again homogenized by stirring for an additional 15 min. Afterwards, the initiator solution (1 wt%) in the amount of 0.06 mol% of the monomer was added and the reaction mixture was heated to 353 K under constant stirring for 6 h. Subsequently, the obtained product was neutralized with a Na_2_CO_3_ solution (3 wt%) and thus converted into the Na^+^ form (60%). The resulting hydrogel was cut into approximately equal discs and placed in excess distilled water to remove unreacted monomer and the sol fraction of the polymers. The synthesized, properly washed-out and dried xerogel, as previously described [29], was kept in a vacuum desiccator until use.

### 4.3. Nicotinamide Loading

The PAM xerogel was loaded with NIAM by the technique of absorption that was described in previous work [29]. In brief, the procedure consists of the following: The predetermined weight of PAM xerogel (1 g) was immersed in excess (250 mL) of a 1 wt% NIAM solution in bidistilled water and left for 24 h at room temperature (293 ± 2 K). Subsequently, the loaded hydrogel was centrifuged in order to remove the excess of solution. Afterward, the loaded hydrogel was gently wiped by tissue paper and left to dry in a thermal oven, first for 4 h at 333 K and then for 2 h at 378 K. The quantity of loaded NIAM was determined by gravimetric method. The used PAM xerogel sample was loaded with a very high amount of NIAM—2 g NIAM per 1 g xerogel.

### 4.4. Kinetics of In Vitro Nicotinamide Release

**Figure 7:**
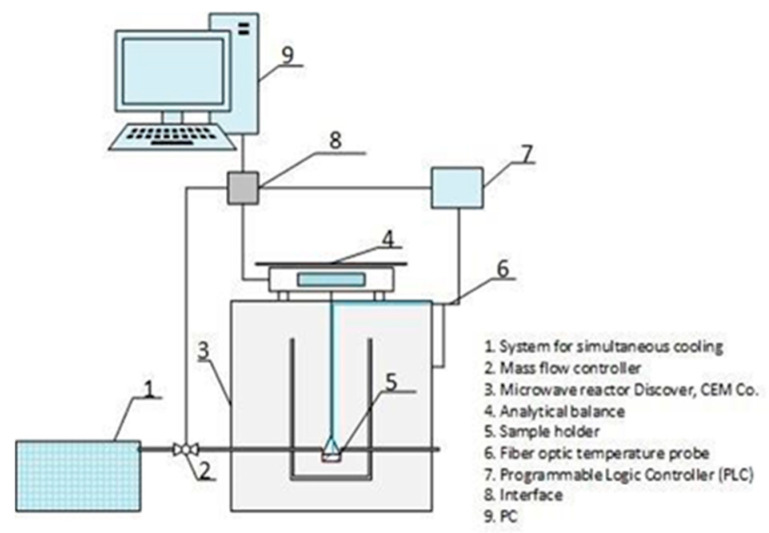
Scheme of the modified microwave device.

The study of the release kinetics of absorbed NIAM under the conditions of SMHC was performed on a modified microwave device—Discovery—produced by CEM corporation, USA, at a working frequency of 2.45 GHz. Figure 7 shows a scheme of the modified microwave device.

A detailed description of the working principle of themodified microwave device is given in the work of B. Koturevic et al. [38]. With an aim to investigate NIAM release kinetics, 0.2 g of the xerogel with absorbed NIAM was placed in a perforated container made of Teflon. The container with the sample was immersed in a microwave reactor performed from borosilicate glass, which was placed in the microwave device. The microwave reactor was previously dispensed with 120 mL of distilled water preheated at desired temperature. At predetermined time intervals, aliquots of the solution were withdrawn from the solution in order to determine the concentration of NIAM in the solution (*C_R_*, g/L). The spectra of the samples were recorded on a UV–Visible Spectrometer, Agilent 8453, USA. The *C_R_* was determined using the values of absorbency at λ = 262 nm (concentration interval C= 0.003–0.03 g/L, molar absorption coefficient (a) a = 5291.5 L/cm mol). For each temperature, measurements of at least three samples were performed and the mean values were used. The amount of the released NIAM (*M_t_*) was calculated by Equation (8):

(8) $$M_{t}=C_{R}V_{s}$$

where *V_S_* is volume of the solution.

The degree of the released NIAM (*α*) was calculated by using Equation (9):

(9) $$\alpha =\frac{M_{t}}{M_{\infty }}$$

where *M_t_* is amount of the NIAM released in time *t* and *M_∞_* is weight of NIAM absorbed on the hydrogel.

### 4.5. Model-Fitting Method

Since the process of drug release from polymer matrices takes place with the contribution of a solid phase, the choice of the kinetics equation for fitting the experimental kinetics data was performed using the model-fitting method [32]. In accordance with the model-fitting approach, the experimentally determined kinetics conversion curves (*α_exp_ = f*(*t*)*_T_*) are transformed to the experimentally normalized conversion curves (*α_exp_* = *f*(*t_N_*)*_T_*), where t_N_ is the so-called normalized time. The normalized time, *t_N_*, was defined by Equation (10):

(10) $$t_{N}=\frac{t}{t_{0.9}}$$

where *t*_0.9_ is time when is achieved *α* = 0.9.

The experimentally normalized conversion curves are then analytically compared with the normalized conversion curves of the theoretical kinetics models. The sum of squares of the deviation (SSD) is used as a measure of derivation of the normalized experimental conversion curves from the theoretical ones. The experimental kinetics data are fitted with the theoretical equation whose SSD minimally deviates from the normalized experimental conversion curves.

## Figures and Tables

**Table 1:** Effect of temperature on the values of *k_M_* and kinetic parameters for SMCH and CH conditions.

T, K	SMCH		CH	
	*k_M_* 10^3^, s^−1^	Kinetic Parameters	*k_M_* 10^4^ s^−1^	Kinetic Parameters
303	8.4		6.7	
313	9.8	*E_a_* = 25.6 kJ/mol	7.3	*E_a_* = 14.1 kJ/mol
318	12	ln(A/s^−1^) = 5.21	8	ln(A/s^−1^) = −1.79
323	13.2		8.7	
328	15.7		9.1	

**Table 2:** Calculated values of T*.

*T*, K	*Z*	T*, K	Δ*T*, K
303	12.6	571	263
313	13.3	599	286
318	14.7	641	323
323	15.3	672	349
328	16.8	721	393

**Table 3:** Effects of heating mode on the values of ΔH* and ΔS*.

Heating Mode	ΔH* (kJ/mol)	ΔS* (J/mol K)
CH	11.84	−259.6
SMHC	23.82	−201.4

**Table 4:** Values of SET’s model parameters.

Parameter	Value
ν (cm^−1^)	274
ε (kJ/mol)	3.31
x	−0.015
